# Non-Alcoholic Fatty Liver Disease Association with Cardiac Arrhythmias

**DOI:** 10.7759/cureus.1165

**Published:** 2017-04-13

**Authors:** Muhammad A Mangi, Hiba Rehman, Abdul M Minhas, Muhammad Rafique, Vikas Bansal, Jonathan Constantin

**Affiliations:** 1 GME Internal Medicine, Orange Park Medical Center; 2 Internal Medicine, Orange Park Medical Center; 3 Anesthesiology, Liaquat National Hospital; 4 Critical Care Medicine , Mayo Clinic Jacksonville, Fl; 5 Cardiology, Orange Park Medical Center

**Keywords:** nafld, conduction defects, atrial fibrillation, prolong qtc interval, ventricular arrythmia

## Abstract

Non-alcoholic fatty liver disease (NAFLD) has become a public health burden all over the world. A significant percentage of the patients with NAFLD have a co-existing metabolic syndrome that is a risk factor for cardiovascular disease. Clinical as well as epidemiological research shows that NAFLD is not simply related to liver-related morbidity and mortality but is also associated with an elevated risk of coronary heart disease (CHD), irregularities of cardiac function as well as cardiac structure, valvular heart disease, and arrhythmias. Animal studies suggest that NAFLD by itself exacerbates systemic/hepatic insulin resistance, leads to atherogenic dyslipidemia and generates a number of pro-inflammatory, pro-coagulant and profibrogenic mediators which play an essential role in the pathophysiology of cardiac abnormalities including arrhythmias. Hence, it is suggested that the patients with NAFLD may derive benefit from intensive monitoring and treatment methods to reduce the risk of CHD along with other cardiac/arrhythmic complications. The intent of this clinical review is to sum up the quickly increasing body of evidence that provides support for a robust relationship between NAFLD and cardiac arrhythmias and to present the putative biological mechanisms underlying this correlation.

## Introduction and background

Nonalcoholic fatty liver disease (NAFLD) is a fatty infiltration of the liver in the absence of alcohol use or liver infection. NAFLD is a common disorder, affecting almost 30%-35% of the US and western population [1-4]. NAFLD is the third most common indication for the liver transplant and is proposed to be the most common cause of end-stage liver disease and liver transplant in the next 10 years [5-6]. The increasing evidence over the past decade suggests that NAFLD is a multisystem disease, it not only damages the liver but also affects the cardiovascular system and causes structural and functional changes in the heart and vessels, leading to increased cardiac-related morbidity and mortality [7-9]. NAFLD is the emerging cause of cardiac arrhythmias. Several arrhythmias are found in association with NAFLD such as atrial fibrillation, QTc (corrected QT) prolongation and ventricular arrhythmias, which can predispose these patients to sudden cardiac death. Here, we provide an extensive literature search of association between NAFLD and various cardiac arrhythmias.

## Review

### Association of NAFLD with atrial fibrillation

Previously, many studies have shown a strong association between NAFLD and coronary heart disease [10-13]. Some studies have also demonstrated fatal and nonfatal cardiovascular events [14-17]. Additional abnormalities include structural changes in the heart and cardiac metabolism [18-22]. Comparatively, fewer studies have evaluated the association of NAFLD with the electrical abnormalities of the heart. 

Sinner, et al. analyzed 3,744 adult patients from the Framingham Heart Study and described the association of elevated transaminase level with atrial fibrillation (AF). He found out that the elevated aspartate transaminase (AST) and alanine transaminase (ALT) levels were independently associated with the increased risk of AF over a 10 year follow-up period (Table 1). In this group of the population, they excluded the patients with clinical heart failure [23]. This association was consistent even after adjusting for other risk factors of AF. In an observational study of 702 diabetic patients, Targher, et al. reported a significant association of NAFLD with the prevalence of AF. In this study, NAFLD was diagnosed with the ultrasound (US) [1]. In another study, Targher and colleagues demonstrated that the incidence of AF is high in the patients with NAFLD over a 10 year follow-up period. This association was independent of age, sex, body metabolic index (BMI), and hypertension [24]. Additionally, Karajgmaki, et al. in their prospective study further confirmed the association of NAFLD with AF [25].

**Table 1:** Studies showing association of NAFLD with atrial fibrillation NAFLD: non-alcoholic fatty liver disease.

Author	Year Published	Study Design	Number of patients	NAFLD Diagnosis	Study Measure	Main Findings
Sinner, et al. [23]	2013	Prospective cohort study	3744 patients with no clinical heart failure (from the Framingham Heart Study original and offspring cohorts)	Liver enzymes	Incidence of atrial fibrillation -10 years of follow-up	Elevated transaminases are independently associated with the increased incidence of atrial fibrillation.
Targher, et al. [1]	2013	Cross-sectional study	Total: 702 NAFLD: 514 Non-NAFLD: 188	Ultrasound	Prevalence of persistent or permanent atrial fibrillation	NAFLD is associated with an increased prevalence of persistent or permanent atrial fibrillation in patients with Type 2 diabetes, independently of several clinical risk factors for atrial fibrillation.
Targher, et al. [24]	2013	Prospective cohort study	Total: 400 NAFLD: 281 Non-NAFLD: 119	Ultrasound	Incidence of atrial fibrillation -10 years of follow-up	NAFLD is associated with the increased incidence of atrial fibrillation in patients with type 2 diabetes even after adjustment for important clinical risk factors for atrial fibrillation.
Karajgmaki, et al. [25]	2015	Prospective cohort study	Total; 958 NAFLD: 249 Non-NAFLD: 709	Ultrasound	Risk of atrial fibrillation -Mean follow-up time was 16.3 years	NAFLD is independently associated with the increased risk of atrial fibrillation.

### Association of NAFLD with prolonged QTc interval and ventricular arrhythmias

Research is being done on the association of NAFLD with prolonged QTc interval for the last few years. Liu, et al. showed the presence of cardiac autonomic dysfunction as evident by changes in heart rate variability parameters in the non-diabetic patients with NAFLD [26]. Targher, et al. conducted a study on 460 patients (NAFLD 281, No-NAFLD 179) with diabetes mellitus (DM) to examine the relationship of NAFLD with prolonged QTc (Table 2) [27]. The presence of NAFLD was found to be strongly associated with increased QTc interval. Additionally, Hung and colleagues conducted a large cross-sectional study involving 31,116 patients and demonstrated that the severity of NAFLD was associated with higher risk for QTc prolongation in the patients with and without diabetes mellitus (DM) [28]. This association was independent of age, coronary artery disease (CAD), systolic blood pressure (SBP), metabolic syndrome, body metabolic index (BMI), hemoglobin A1c (HbA1c), aspartate transaminase (AST) and estimated glomerular filtration rate (eGFR). A recent retrospective study showed an association of NAFLD with QTc prolongation on univariate analysis (OR 5.09, 95%CI (2.92-8.86), p-value < 0.0001) [29]. Motvani, et al. retrospectively analyzed 330 outpatients with type 2 DM without pre-existing atrial fibrillation (AF), end-stage renal disease (ESRD) or liver disorder [30]. These patients underwent 24-hour Holter monitoring (Holter Research Laboratory, Helena Montana, United States) for various clinical reasons. They reported that the patients with NAFLD when compared with the patients without NAFLD, had a higher prevalence of > 30 premature ventricular contractions (PVCs) per hour, non-sustained ventricular tachycardia (VT) or both. This association remained consistent after adjusting for age, sex, BMI, smoking, chronic kidney disease, chronic obstructive pulmonary disease, ischemic heart disease, valvular heart disease, serum gamma-glutamyl transferase, medication use and ejection fraction.

**Table 2:** Studies showing association of NAFLD with prolonged QTc interval and ventricular arrhythmia NAFLD: Non-alcoholic fatty liver disease. PVCs: Premature ventricular contractions. VT: Ventricular tachycardia. QTc: Corrected QT.

Author	Year Published	Study Design	Number of patients	NAFLD Diagnosis	Study Measure	Main Findings
Targher, et al. [27]	2014	Cross-sectional study	Total: 400 NAFLD 281 Non-NAFLD 179	Ultrasound	Prevalence of prolonged QTc interval	Presence and severity of NAFLD on ultrasound are strongly associated with increased QTc interval in patients with type 2 diabetes even after adjusting for multiple established risk factors and potential confounders
Hung, et al. [28]	2015	Cross-sectional study	Total: 31116 NAFLD: 12891 Non-NAFLD: 18225	Ultrasound	Prevalence of prolonged QTc interval	The severity of NAFLD is associated with a higher risk for QTc prolongation in the general population with and without diabetes
Mantovani, et al. [30]	2016	Cross-sectional study	Total: 330 NAFLD: 238 Non-NAFLD: 92	Ultrasound	Prevalence of ventricular arrhythmias(non-sustained VT, >30 PVCs/hour, or both)	NAFLD is independently associated with an increased risk of prevalent ventricular arrhythmias in patients with type 2 diabetes

### Association of NAFLD with conduction defects

A study conducted to ascertain the relationship of NAFLD with right bundle branch block (RBBB) showed a positive association [31]. The authors of this study postulated that the patients with RBBB are at a higher risk of developing NAFLD due to passive congestion of the liver. A recent retrospective study described the increase prevalence of conduction defects in the patients with NAFLD (OR 2.38, 95%CI (1.51-3.73), p-value < 0.0001) (Table 3) [29]. The authors of this study postulated that the patients with NAFLD, determined on imaging, are at higher risk of fat deposition in the cardiac muscles and vessels, resulting in the conduction defects.

**Table 3:** Studies showing association of NAFLD with conduction defect RBBB: Right bundle branch block. NAFLD: Non-alcoholic fatty liver disease.

Author	Year Published	Study Design	Number of patients	NAFLD Diagnosis	Study Measure	Main Findings
İşcen, S. [31]	2013	Cross-sectional study	Total: 2200 RBBB: 220 No-RBBB: 1980	Ultrasound	Risk of NAFLD in RBBB	RBBB is associated with an increased risk of NAFLD in young healthy individuals
Mangi, et al. [29]	2017	Case- control study	Total; 700 NAFLD: 408 Non-NAFLD: 292	Ultrasound, CT scan of abdomen	Risk of conduction defects	NAFLD is associated with conduction defects

### Mechanism of NAFLD causing cardiac arrhythmias

The mechanism of NAFLD association with cardiovascular disease (CVD) is not well known, but various hypotheses have been generated for the possible pathogenesis of CVD in the patients with NAFLD. It is debatable if NAFLD is a mere risk marker for the development of CVD or NAFLD is the direct contributor to the development of the disease. Initially, it was thought that the association between NAFLD and CVD/arrhythmias might be due to the fact that they share common risk factors, but more and more studies are being done which show their relation independent of these confounders. It is difficult to unwind this complex relationship as NAFLD and CVD share similar risk factors. NAFLD might be a marker for ectopic fat deposition in myocardium and pericardium. These structural heart changes predispose to cardiovascular events. Previously, a study has shown an association between intrahepatic and myocardial triacylglycerol content and that increased pericardial fat is associated with increased prevalence of atrial fibrillation [32-33]. Experiments have also revealed that adipocytes in retrosternal, epicardial tissue can exert effects on ion currents in rabbit left atria, leading to arrhythmias and other complications [34]. However, it has been hypothesized that NAFLD directly contributes to the development of cardiovascular complications. For instance, it has been proposed that NAFLD is associated with increased production of pro-inflammatory cytokines (C - reactive protein, interleukin - 6 and tumor necrosis factor – alpha), prothrombotic factors (fibrinogen, factor VIII, plasminogen activator inhibitor -1) [35-38]. These markers have been shown to be associated with increased cardiac structural and arrhythmogenic complications, possibly by cardiac structural and electrical remodeling [39-41].

### Treatment of NAFLD

NAFLD is being seen as one of the important risk factors for cardiovascular morbidity and mortality [42-43]. It is plausible that the health care provider should treat the patients with NAFLD aggressively to decrease cardiovascular morbidity and mortality. The lifestyle modifications, including weight loss, dietary modifications, and physical activity are the first line treatment for NAFLD and have shown to improve the outcomes [44-45]. The patients should be given pharmacotherapy if nonalcoholic steatohepatitis (NASH) is present [46]. Treatment of DM, hyperlipidemia, and obesity not only improve the hepatic histology but also improve the cardiovascular outcomes [47]. Concurrent management of hypertension does not have a major impact on liver histology but improves cardiovascular morbidity [47]. Additionally, thiazolidinediones (pioglitazone and rosiglitazone) and liraglutide are approved for the management of NAFLD [48-49].

## Conclusions

The role of NAFLD in cardiovascular complications has been extensively researched. Previous studies have shown the association of NAFLD with cardiac structural, metabolic and functional changes. Fewer studies have been conducted on the association with cardiac arrhythmias. These studies have shown that NAFLD has arrhythmogenic potential which requires further testing and confirmation. The pathophysiological mechanism of NAFLD is not very well understood. More research is needed to establish the pathways that are involved in the development of cardiovascular complications. We believe that NAFLD is a promising field of research to explore the pathways involved in the causation of arrhythmias that could possibly provide a therapeutic target for treatment and help in the prevention of cardiac remodeling and electrophysiological abnormalities.
